# Inhibition of HOX/PBX dimer formation leads to necroptosis in acute myeloid leukemia cells

**DOI:** 10.18632/oncotarget.20023

**Published:** 2017-08-07

**Authors:** Raed A. Alharbi, Hardev S. Pandha, Guy R. Simpson, Ruth Pettengell, Krzysztof Poterlowicz, Alexander Thompson, Kevin Harrington, Mohamed El-Tanani, Richard Morgan

**Affiliations:** ^1^ Department of Laboratory Medicine, Faculty of Applied Medical Sciences, Albaha University, Albaha, Saudi Arabia; ^2^ Faculty of Health and Medical Sciences, University of Surrey, Guildford, UK; ^3^ St. George’s, University of London, London, UK; ^4^ Institute of Cancer Therapeutics, University of Bradford, Bradford, UK; ^5^ Division of Cancer and Stem Cells, Centre for Biomolecular Sciences, University of Nottingham, Nottingham, UK; ^6^ Targeted Therapy Team, Chester Beatty Laboratories, Institute of Cancer Research, London, UK

**Keywords:** acute myeloid leukemia, HOX, HXR9, necroptosis, protein kinase C

## Abstract

The HOX genes encode a family of transcription factors that have key roles in both development and malignancy. Disrupting the interaction between HOX proteins and their binding partner, PBX, has been shown to cause apoptotic cell death in a range of solid tumors. However, despite HOX proteins playing a particularly significant role in acute myeloid leukemia (AML), the relationship between HOX gene expression and patient survival has not been evaluated (with the exception of *HOXA9*), and the mechanism by which HOX/PBX inhibition induces cell death in this malignancy is not well understood. In this study, we show that the expression of *HOXA5*, *HOXB2*, *HOXB4*, *HOXB9*, and *HOXC9*, but not *HOXA9,* in primary AML samples is significantly related to survival. Furthermore, the previously described inhibitor of HOX/PBX dimerization, HXR9, is cytotoxic to both AML-derived cell lines and primary AML cells from patients. The mechanism of cell death is not dependent on apoptosis but instead involves a regulated form of necrosis referred to as necroptosis. HXR9-induced necroptosis is enhanced by inhibitors of protein kinase C (PKC) signaling, and HXR9 combined with the PKC inhibitor Ro31 causes a significantly greater reduction in tumor growth compared to either reagent alone.

## INTRODUCTION

The molecular mechanisms underlying the pathogenesis of acute myeloid leukemia (AML) have been extensively studied, and are known to involve members of the HOX family of transcription factors, both as partners in chimeric fusion proteins, and also in their wild type form [1]. Whilst these roles are reflected in the relationship between the expression of individual *HOX* genes and clinicopathological factors such as disease subtype and patient survival [2], the role of HOX proteins in the survival of AML cells has proved difficult to assess as many have redundant functions, which makes a conventional knock down experiment difficult to interpret. For example, knocking down the expression of either *HOXA6* or *HOXA9* alone has little effect on AML cells, but their double knock-down induces cell death and also increases their sensitivity to cytarabine [3]. An alternative strategy to targeting HOX proteins is to inhibit their interaction with the PBX co-factor, which can be achieved using a short, cell-penetrating peptide (HXR9) that mimics the conserved hexapeptide in HOX proteins responsible for PBX binding [4]. HXR9 has been shown to induce apoptosis in a range of solid cancers, both *in vitro* and *in vivo*, including those of the lung [5], breast [6] and prostate [7], and melanoma [8]. It has also been shown to be cytotoxic for malignant B cells [9], and a number of AML cell lines [10]. However, the mechanism of HXR9 induced-cell death is not well understood in hematological malignancies. In this study we evaluated the relationship between *HOX* gene expression and overall survival, and the mechanism by which HXR9 causes cell death in AML. Our findings indicate that HXR9 induces necroptosis, rather than apoptosis, and that its cytotoxicity can be greatly enhanced by inhibition of protein kinase C (PKC).

## RESULTS

**Figure 1:**
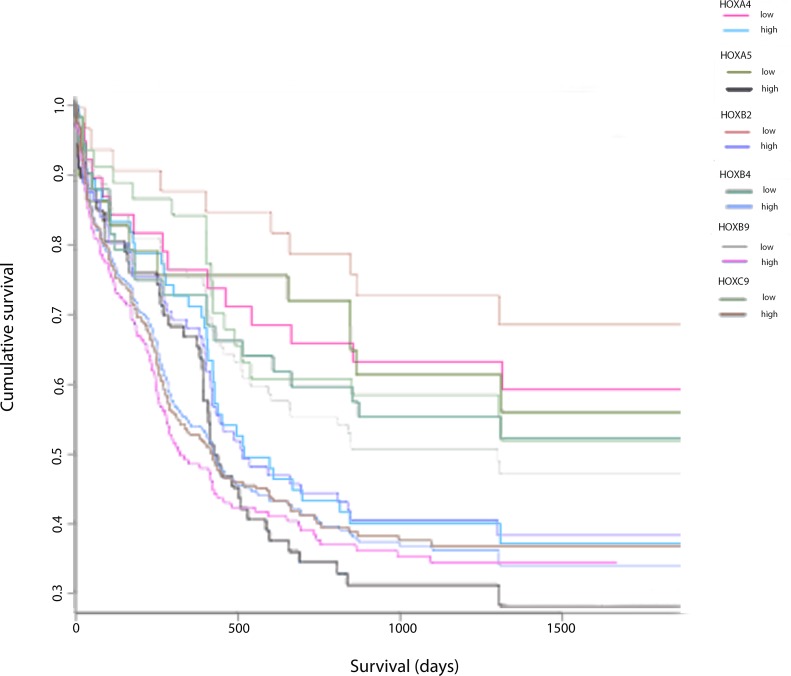
Association of expression of ***HOX*** genes in combination with AML patient survival data Kaplan-Meier plots of the cumulative proportion of patients surviving in the AML dataset (*n* = 269) from the Gene Expression Omnibus database GSE23312 in patients with a low level and a high level of expression of each specified *HOX* gene.

Despite the public availability of large datasets relating *HOX* gene expression to survival in AML, relatively little has been reported on the relationship between the expression of individual *HOX* genes and survival. We therefore analyzed the relationship between survival and expression of *HOX* genes that encode proteins capable of binding to the HXR9 target, PBX, amongst a cohort of 269 patients from the Gene Expression Omnibus (GEO) database [11]. This revealed that a number of *HOX* genes were significantly related to survival in AML, including *HOXA5* (*p* = 0.03), *HOXB2* (*p* = 0.002), *HOXB4* (*p* = 0.037), *HOXB9* (*p* = 0.001), and *HOXC9* (*p* = 0.007) (Figure 1), whilst *HOXA4* (*p* = 0.067) and *HOXA9* (*p* = 0.06) showed borderline significance. In contrast, the expression of a number of other *HOX* genes including *HOXA1* (*p* = 0.242), *HOXA2* (*p* = 0.595), *HOXA3* (*p* = 0.407), *HOXA7* (*p* = 0.529), *HOXB3* (*p* = 0.783), *HOXB5* (*p* = 0.979), *HOXB6* (*p* = 0.246), *HOXB7* (*p* = 0.996), *HOXB8* (*p* = 0.74), and *HOXC6* (*p* = 0.876) were not related to patient survival (data not shown).

**Figure 2:**
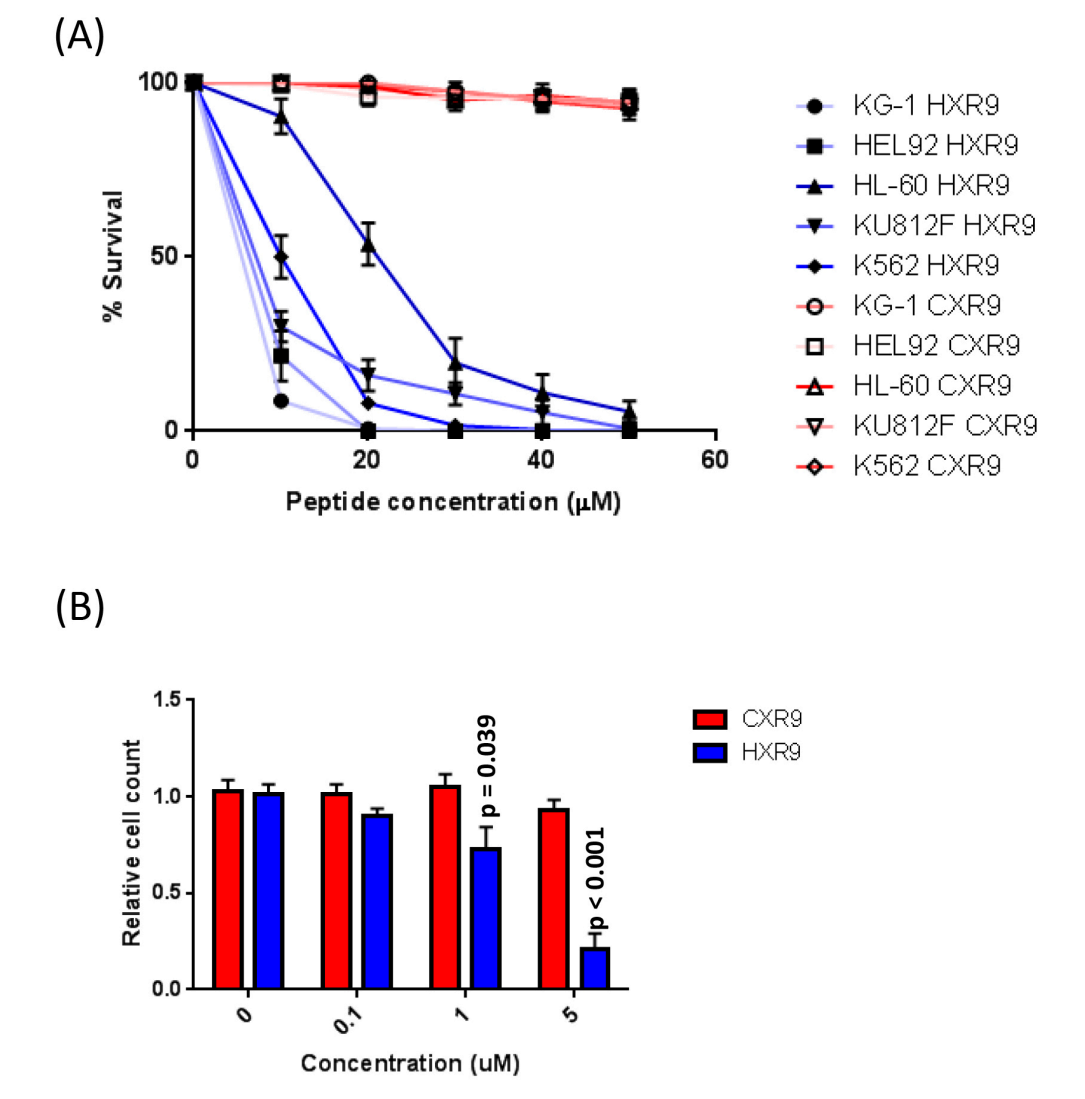
**A.** IC50 survival curves for AML-derived cell lines treated with HXR9 or CXR9. **B.** Proliferation of primary AML cells treated with varying concentrations of HXR9 or CXR9. Each value is the mean of 3 independent repeats, error bars show the SEM.

In order to evaluate the molecular mechanisms underlying the cytotoxicity of HXR9 in AML cells, we determined the sensitivity of a number of AML-derived cell lines and primary AML cells. Three of the cell lines were derived from primary AML (KG1, HEL 92.1.7, and HL-60) and 2 from secondary AML (KU812F, and K562). The IC50s of cell killing by HXR9, as determined using an LDH assay, were 4.5, 6.1, 16.9, 9.1, and 10.4 μM, respectively (Figure 2A). None of these cell lines were sensitive to CXR9, an inactive variant of HXR9 that differs from it by only a single amino acid [7]. In order to test the effect of HXR9 on primary AML cells we isolated cells from the peripheral blood of AML patients and used a proliferation assay to evaluate the response to HOX/PBX inhibition. This revealed that HXR9 can significantly reduce the proliferation of primary AML cells at a concentration < 1 μM (Figure 2B), which is considerably lower than for other primary cancer cells isolated from solid malignancies [8].

**Figure 3:**
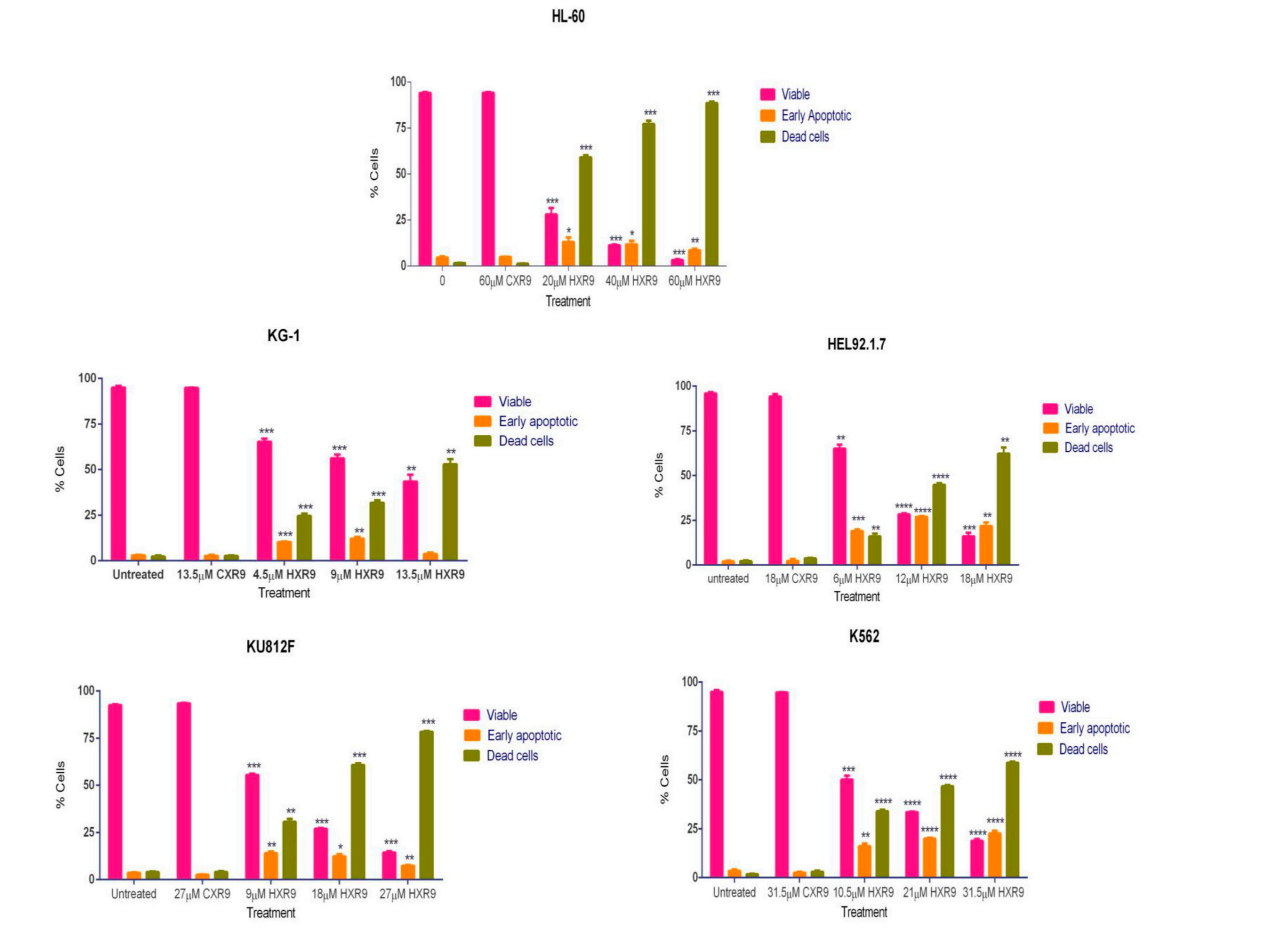
Annexin assay for detection of cell death in AML-derived cell lines Cells were treated with the IC_50_, 2xIC_50_, or 3xIC_50_ of HXR9, and with a concentration of CXR9 equivalent to the highest concentration of HXR9 for 2 hours. Cells were then stained with annexin V and 7-AAD and analysed by flow cytometry. The number of early apoptotic and dead cells increased with increasing HXR9 concentrations. Results are expressed as the mean of 3 separate experiments and error bars show the SEM. *p < 0.05, **p < 0.01, ***p < 0.001 and ****p < 0.0001 with respect to untreated cells.

**Figure 4:**
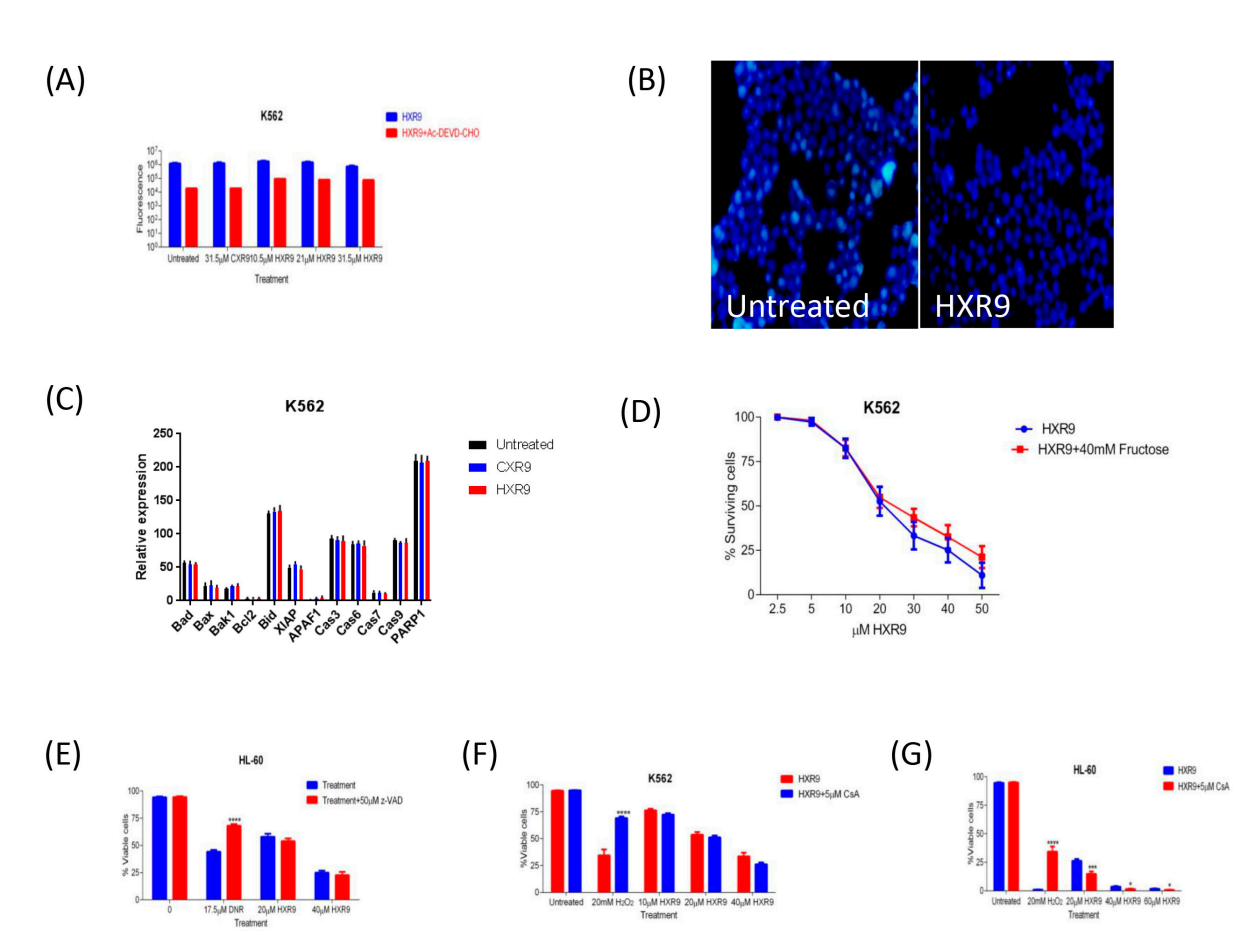
**A.** Inhibition of apoptosis by Ac-DEVD-CHO in AML K562 cells using Z-DEVD-R110. Cells were treated with IC_50_, 2xIC_50_, 3xIC_50_ of HXR9, with an equivalent CXR9 to the highest concentration of HXR9 or untreated (control) for 2 hours. The cells were then lysed and the Z-DEVD-R110 substrate was added. The results were normalised to lysis buffer alone. There was no statistical difference in caspase-3 activity between both HXR9- or CXR9-treated cells and untreated control cells. Graphs show the mean of 3 independent experiments and error bars show the SEM. **B.** Nuclear staining of K562 cells either untreated or incubated for 2 hours with 10 μM HXR9. **C.** Analysis of transcriptional changes of several pro- and anti-apoptotic genes upon HXR9 treatment. K562 cells were treated with the IC_50_ of HXR9, CXR9 or untreated (control) for 2 hours and gene expression was analysed using RT-PCR. There was no significant change in any of the analysed genes. Results are presented as a ratio with *β-actin* (x10 000). Statistical analysis was performed using student's *t-test* by comparing the relative expression of a gene of interest in HXR9 or CXR9 treated cells to its counterpart in untreated cells. Graphs show the mean of 3 independent experiments and error bars show the SEM. **D.** The effect of ATP depletion on HXR9 cytotoxicity. Cells were pre-incubated with or without 40 mM fructose and then with a range of HXR9 concentrations (2.5 μM - 50 μM) for 2 hours prepared in media either with or without 40 mM fructose, red and blue curves, respectively. HXR9 cytotoxicity was then measured by assessing LDH enzyme activity in cell-free supernatants. ATP depletion did not inhibit HXR9-induced death. Graphs show the mean of 3 independent experiments and error bars show the SEM. **E.** General inhibition of caspase activity in HXR9 treated AML cell lines by z-VAD-FMK. Cells were pre-treated either with or without 50μM z-VAD-FMK for 1 hour, and then treated with the IC_50_, or 2xIC_50_ of HXR9 for 2 hours or with 17.5 μM DNR for 24 hours, which was used as a positive control. There was no statistical difference in terms of sensitivity to HXR9 between pre-treated cells with or without 50μM z-VAD-FMK. Graphs show the mean of 3 independent experiments and error bars show the SEM. ****p < 0.0001 with respect to z-VAD-FMK untreated cells. **F.** and **G.** Effect of CsA on HXR9 cytotoxicity. Cells were pre-treated with or without 5μM CsA for 1 hour, and then with the IC_50_, 2xIC_50_, 3xIC_50_ of HXR9, 20 mM H_2_O_2_ (positive control), or were left untreated (negative control) for 2 hours with or without 5 μM CsA. The cells were then stained with annexin V and 7-AAD and analysed by flow cytometry. There was no statistical difference in the proportion of viable cells between K562 cells **F.** incubated with or without 5μM CsA, while incubation with 5μM CsA led to a significant decrease in proportion of viable HL-60 cells **G.**. Graphs show the mean of 3 independent experiments and error bars show the SEM. *p < 0.05, ***p < 0.001 and ****p < 0.0001 with respect to 5 μM CsA untreated cells.

**Figure 5:**
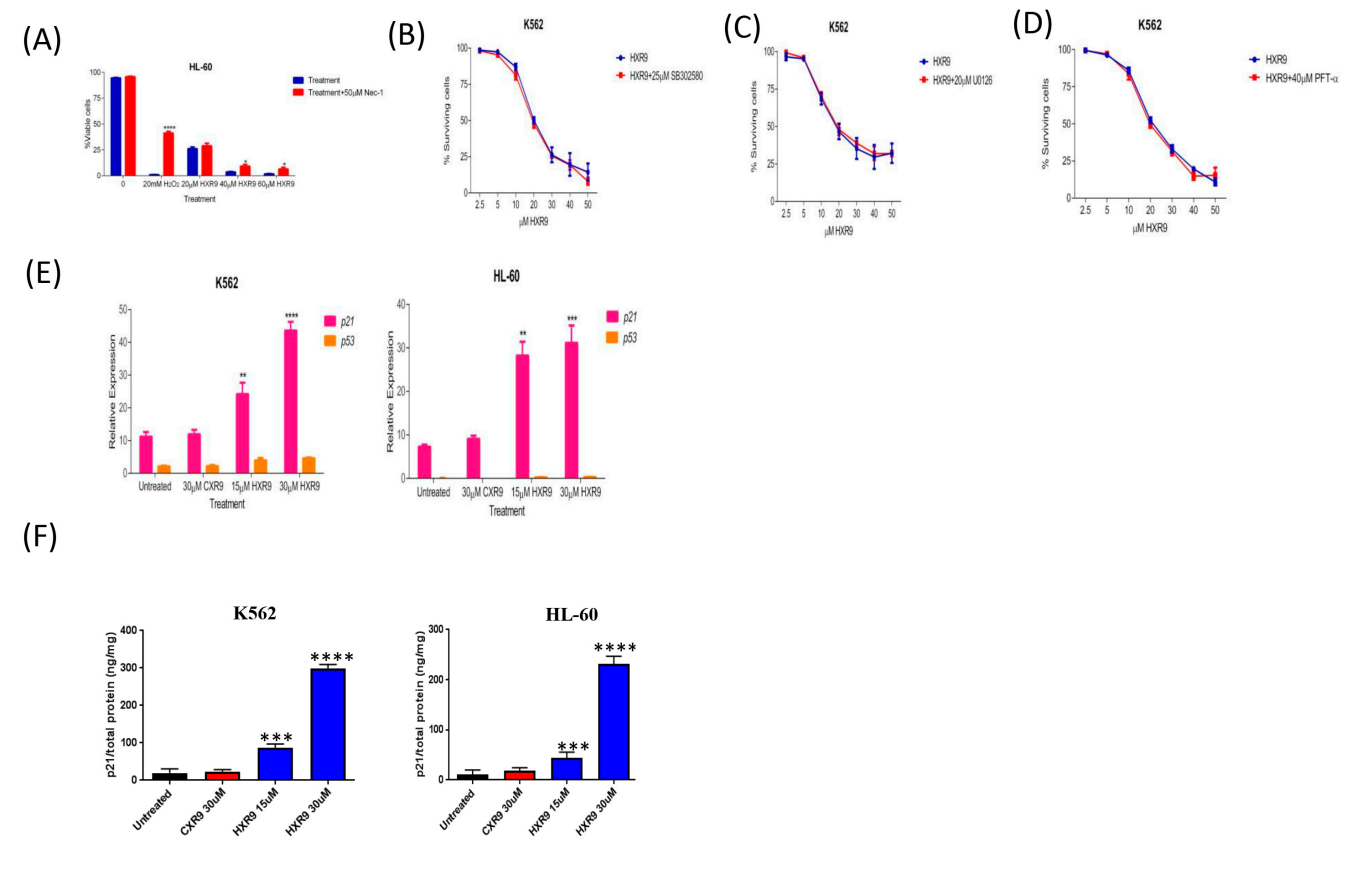
Evaluation of potential cell death pathways **A**. Inhibition of RIP1 rescues HL-60 cells from HXR9-mediated cell killing. RIP1 was inhibited using 50 μM Nec1. Hydrogen peroxide was used as a positive control as it has previously been shown to induce RIP1-dependent cell killing. **B.** Cells were pre-treated with or without 25 μM of the p38 inhibitor SB302580 for 1 hour, and then with a titration of HXR9 prepared in media with or without 25 μM SB302580, red and blue curves, respectively. The cytotoxicity of HXR9 was analysed by measuring LDH enzyme activity in cell-free supernatants. Pre- and co-treatment of cells with 25 μM SB302580 did not affect the cytotoxicity of HXR9. Statistical significance was tested using the student's *t-test*. Graphs show the mean of 3 independent experiments and error bars show the SEM. **C.** Cells were pre-treated with or without 20 μM of the MEK/ERK inhibitor U0126 for 1 hour, and then with increasing concentrations of HXR9 prepared in media with or without 20 μM U0126, red and blue curves, respectively. The proportion of surviving cells was then measured using the LDH assay. Pre- and co-treatment of cells with 20μM U0126 did not prevent HXR9 cytotoxicity. Graphs show the mean of three independent experiments and error bars show the SEM. **D.** The effect of blocking p53 protein on HXR9 cytotoxicity. Cells were pre-treated with or without 40 μM PFT-α for 1 hour, and then for 2 hours with a titration of HXR9 prepared in media supplemented with or without 40 μM PFT-α, red and blue curves, respectively. Graphs show the mean of 3 independent experiments and error bars show the SEM. **E.** HXR9 treatment of K562 and HL-60 cells leads to a significant increase in expression of the *p21* tumor suppressor gene. *p21* expression was measured using quantitative PCR after 2 hours of incubation with the IC50 concentration of HXR9 for each cell type. **F.** p21 protein expression was measured using an ELISA with the same concentrations of HXR9.

**Figure 6:**
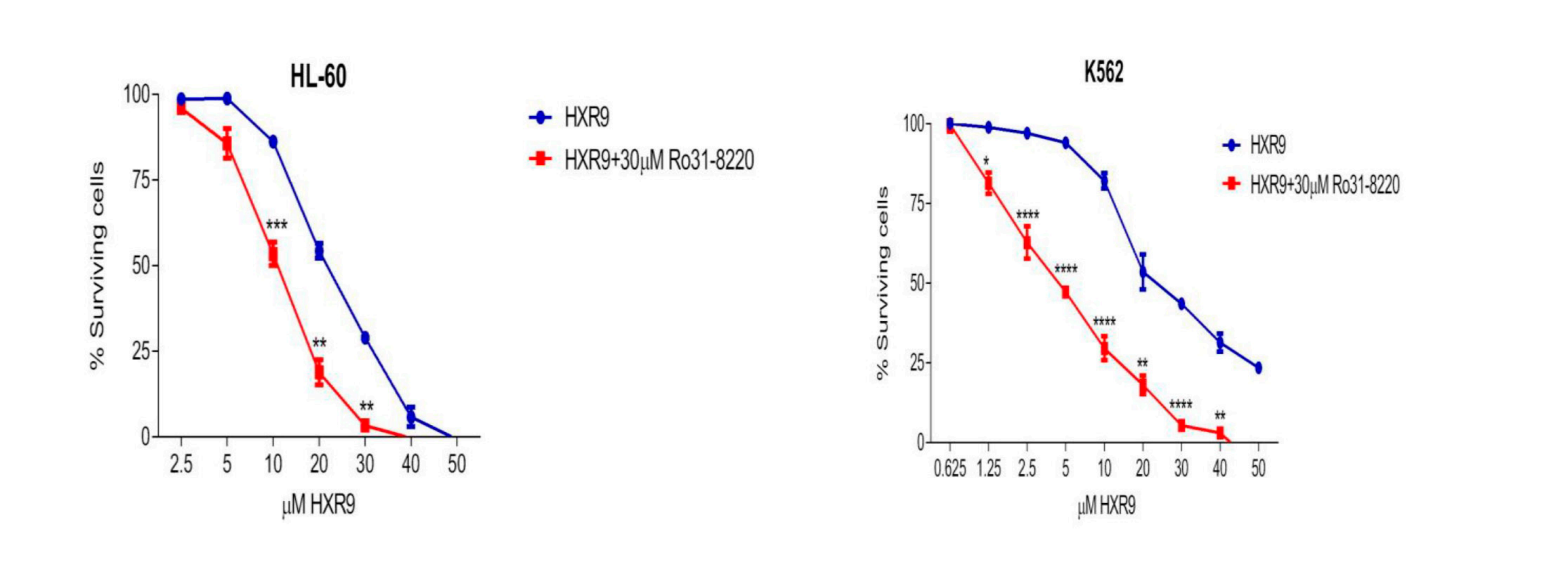
Inhibition of PKC sensitizes cells to killing by HXR9 PKC was inhibited using Ro31-8220, reducing the IC50 of HXR9 to 5 μM for K562 cells, and to 10.7 μM for HL-60 cells.

We investigated whether these cells underwent apoptosis after HXR9 treatment. Although changes in the plasma membrane consistent with apoptosis were apparent in all of these cell lines (Figure 3), which concurs with previous findings [10], this is not in itself an absolute indication that cells have undergone apoptosis, as these membrane changes can also occur during necrosis [12]. Indeed, further studies with the K562 and HL-60 cell lines revealed no evidence of other, more definitive changes associated with apoptosis including caspase-3 activation (Figure 4A) or nuclear fragmentation (Figure 4B). We also measured the expression of a number of genes involved in apoptosis using qRT-PCR (Figure 4C). This analysis revealed that HXR9 did not cause transcriptional changes in the initiator *caspase-9* or its partner *Apaf1* that together form an apoptosome complex with cytochrome C (cyt C) [13], nor in the executioner *caspases-3*, *-6* and *-7*, which are key mediators of apoptosis [14], nor the pro-apoptotic members of the Bcl-2 family including *Bad*, *Bax*, *Bak1* and *Bid*, nor the anti-apoptotic member *Bcl-2*, which mediate mitochondrial cell death [15]. In addition, neither the transcription of *PARP1* nor *XIAP* was affected by HXR9 treatment. Furthermore, cell death was not dependent on ATP (Figure 4D), and could not be reversed by the pan-caspase inhibitor z-VAD-FMK (Figure 4E). We therefore explored the possibility of necrotic cell death. K562 cells could be rescued from HXR9-mediated cell killing using CsA (Figure 4F), an inhibitor of mitochondrial necrosis that targets the CypD protein, although only a limited rescue was achieved in HL-60 cells (Figure 4G) [16]. However, inhibition of the RIP1 kinase using its inhibitor Nec-1 [17] resulted in a significant rescue of K562 and HL-60 cells from HXR9-mediated cytotoxicity (Figure 5A). RIP1 is a central component of the necroptosis pathway [18], suggesting that this might play a key role in HXR9-induced cell death. We also explored further molecular pathways that might influence necroptosis. Inhibition of signaling through p38 (Figure 5B) and MEK/ERK (Figure 5C) had no effect on HXR9 cytotoxicity, nor did the inhibition of the p53 tumor suppressor protein (Figure 5D), although HXR9 treatment of both K562 and HL-60 cells resulted in a significant increase in expression of the *p21* tumor suppressor gene at both the RNA (Figure 5E) and protein (Figure 5F) level. Furthermore, inhibition of PKC also significantly sensitized cells to HXR9 (Figure 6).

**Figure 7:**
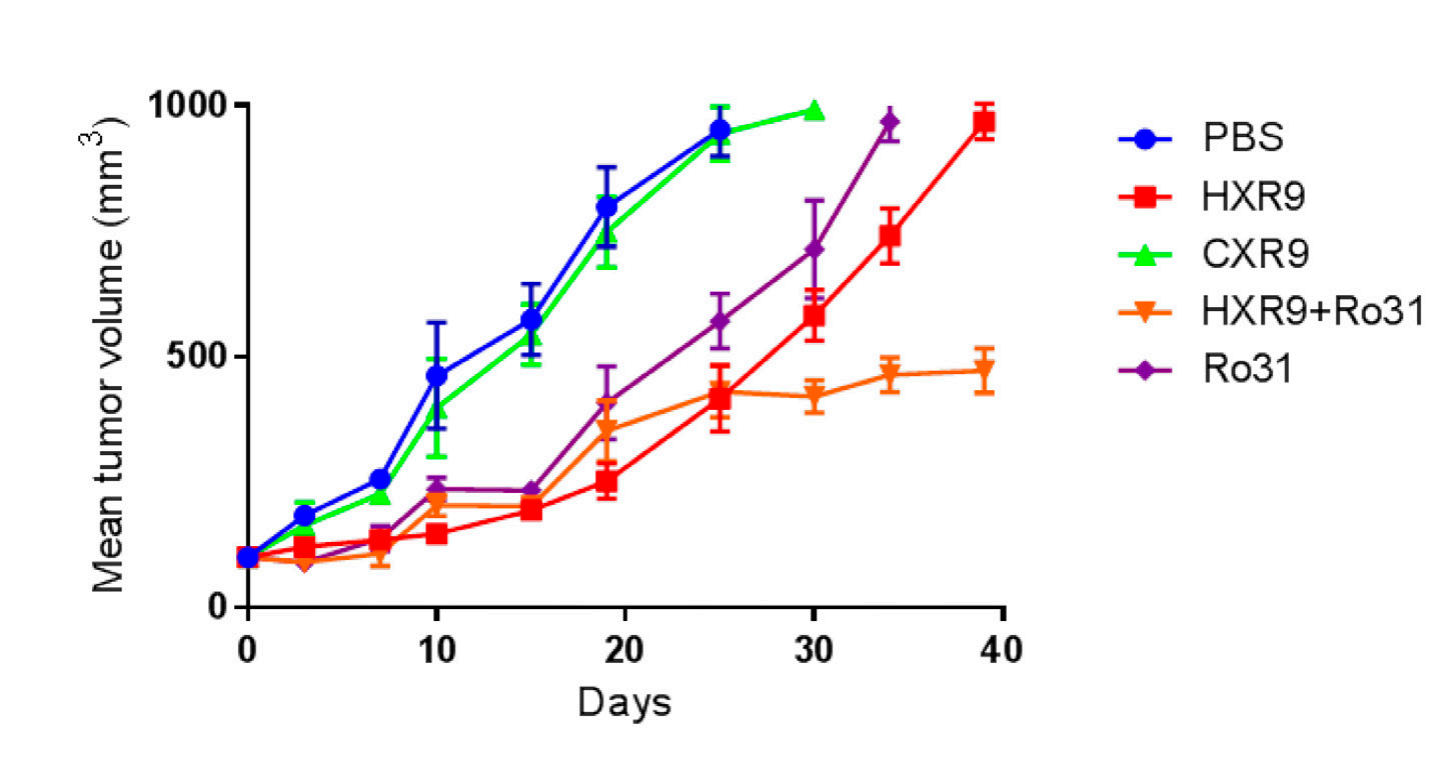
Inhibition of K562 tumor growth in a mouse xenograft model Tumors were initiated by subcutaneous injection of K562 cells in to the flank of each mouse and were treated intratumourally with HXR9 or CXR9 at a dose of 50 mg/Kg, or Ro31 at a dose of 10 mg/Kg, or both HXR9 and Ro31, every 3 days.

In order to test these findings *in vivo*, we used a previously described murine model of AML based on a xenograft flank tumor model using K562 cells. Treatment with HXR9 significantly reduced tumor growth compared to vehicle (PBS) alone or CXR9, and combining HXR9 with the PKC inhibitor Ro31 resulted in a significantly longer delay in tumor growth (Figure 7).

## DISCUSSION

The *HOX* genes have previously been shown to have a key role in the development of a malignant phenotype in AML, although this has principally been related to their incorporation in chimeric oncogenes formed by chromosomal breakage and rearrangement [1]. Less is known of their role in their wild type from, although the overexpression of a number of *HOX* genes is important for cell survival and drug resistance [3]. In this study, we examined the relationship between overall survival and the expression of individual *HOX* genes in AML. It was previously shown that high *HOXA9* expression was associated with a poor outcome in this malignancy [2], although in the larger cohort examined here this relationship was only of borderline significance. The reason for this difference is unclear, although it may reflect the high level of functional redundancy amongst *HOX* genes with respect to proliferation and survival. Indeed, we found that the expression of a number of other *HOX* genes in AML showed a strong association with survival, including *HOXA5*, which has been shown to be upregulated by the H3K79 methyltransferase hDOT1L in MLL-AF10-mediated leukaemogenesis [19].

The strong relationships between the elevated expression of multiple *HOX* genes in AML and patient survival support the possibility that HOX proteins could be therapeutic targets in this malignancy, and this is supported further by our findings that inhibition of HOX/PBX dimers cause cell death in AML-derived cell lines and primary AML cells. Taken together, our findings point to a mechanism of HXR9-mediated cell death that depends not on apoptosis, but instead on necroptosis, and which can be blocked by PKC signaling. Necroptosis is considered to be a regulated form of necrosis that in some respects parallels apoptosis, as it can be triggered by the same external stimuli [18]. It is a major mechanism of cell death in chronic lymphocytic leukaemia (CLL) [20], induced by drugs that target Bcl-2 [21] and the proteasome [22]. In addition, the induction of necroptosis in AML cells has been used as a strategy to overcome resistance to apoptosis, through the combined use of a Smac mimetic that inhibits XIAP and agents that promote DNA demthylation [23]. It has also been shown to mediate the killing of AML cells by a diphtheria toxin-GMCSF conjugate [24]. Despite this, the lack of any apoptosis following HXR9 treatment in these AML-derived cell lines is curious, as some solid malignancies have been shown to undergo apoptosis and can, for example, be rescued by the inhibition of caspase activity [8, 25].

**Figure 8:**
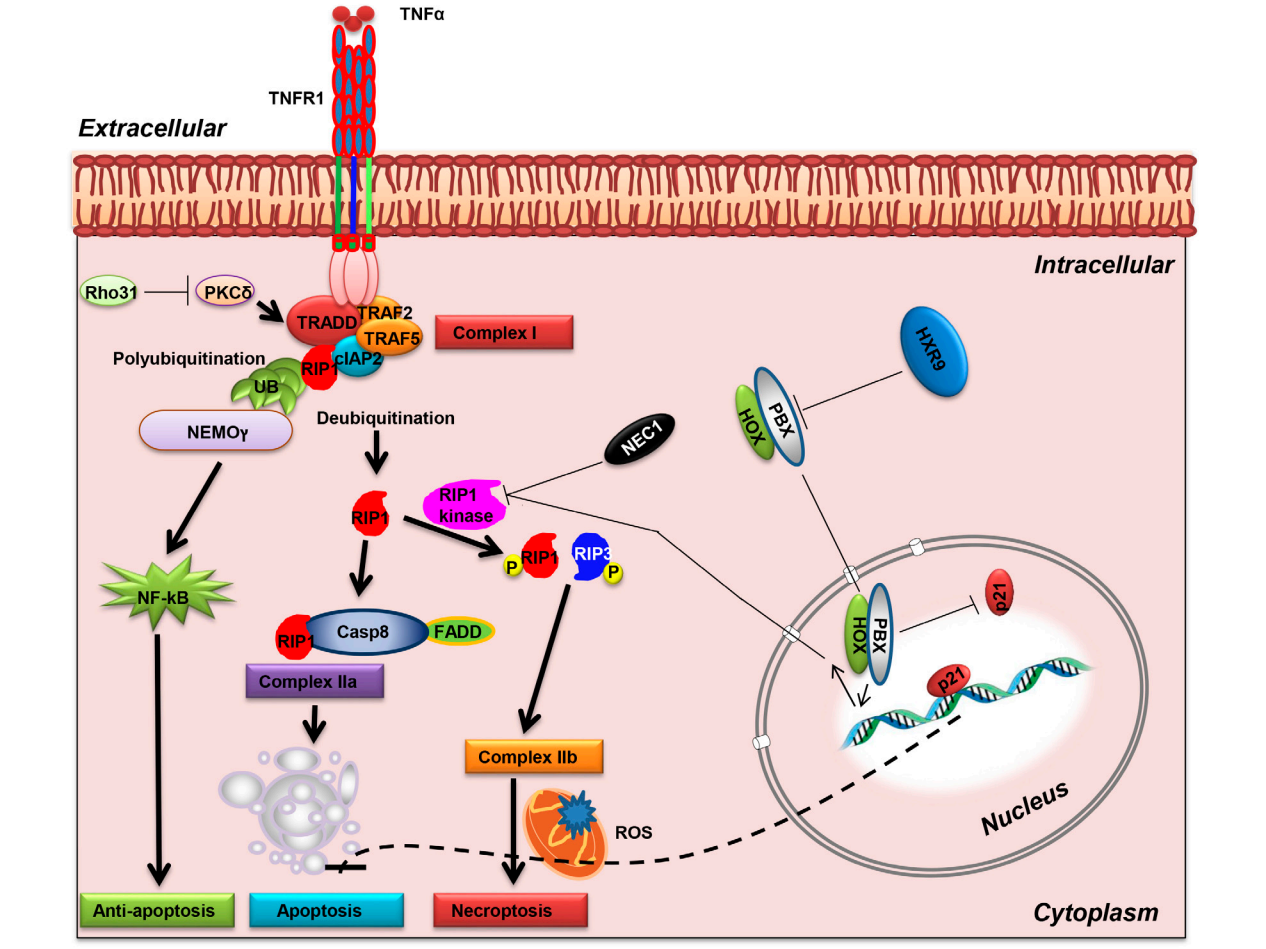
Activation of RIP1-mediated necroptosis TNF-α binding to TNFR1 can activate NF-κB activation, apoptosis, or necroptosis. Phosphorylation of TNFR1 favours TRADD recruitment that in turn leads to RIP1 ubiquitination (complex I), and the subsequent activation of the NF-κB pathway *via* NEMO. Inhibition of PKC delta leads to the deubiquitination of RIP1 and the formation of either complex IIa with FADD and caspase-8, or, if RIP1 is phosphorylated, the formation of a necrosome with phosphorylated RIP3 (complex IIb), leading to necroptosis. A dimer between HOX and PBX proteins represses the necroptosis pathway, possibly through inhibition of RIP kinase. HOX/PBX dimers also repress p21 expression, which might in turn block apoptosis (dotted line). Abbreviations: PKC, protein kinase C; NEMO, NF-kappa-B essential modulator; RIP1, Receptor-interacting protein 1; cIAP2, Cellular inhibitor of apoptosis-1; TRAF, TNF receptor associated factors; FADD, Fas-associated protein with death domain; TRADD, Tumor necrosis factor receptor type 1-associated death domain protein.

PKC has not previously been show to directly interact with the necroptosis pathway, although signaling through PKC is known to promote the transcription of pro-survival genes, and to be a potential target in CLL [26]. Furthermore, the delta-PKC isotype was previously identified as a critical regulator of TNF signalling in adherent neutrophils. Delta PKC associates with tumour necrosis factor receptor-1 (TNFR-1) in response to TNF binding whereupon it promotes receptor serine phosphorylation and consequently enhances binding of TNFR-1-associated death domain protein (TRADD) to TNFR-1 and formation of a TNFR1-TRADD-RIP1 complex [27]. The formation of this complex in turn results in the polyubiquitination of RIP1 and activation of NF-kB signalling, which promotes cell survival. Conversely, disruption of this complex leads to RIP deubiquitination, and its subsequent recruitment to a complex with either Caspase 8, leading to apoptosis, or its phosphorylation by RIP1 kinase followed by binding to RIP3 and the induction of necroptosis (Figure 8) [28]. Our findings indicate that the latter pathway is important as an inhibitor of RIP1 kinase, but not a global caspase inhibitor, significantly reduced the sensitivity of AML cells to killing by HXR9.

In addition to inhibiting necroptosis, our findings indicate that HOX/PBX dimers block p21 expression in AML cells at both the RNA and protein level. The relationship between p21 and apoptosis is complex and varies between cell types: p21 can promote apoptosis in some contexts but prevent apoptosis in others [29]. The lack of apoptosis after HXR9 treatment might be a result of increased p21 expression (Figure 8), although further dissection of this complex molecular pathway is needed in order to confirm this.

The synergistic interaction between HXR9 and PKC-mediated signaling is particularly noteworthy, as it points to possible combinatorial approaches when targeting AML using HOX/PBX inhibitors, and indeed this was supported by results obtained using a murine model. Furthermore, the strong relationship between survival in AML and the expression of a number of *HOX* genes indicates that targeting HOX function *via* the disruption of HOX/PBX dimers could be a useful therapeutic target in this disease, and that *HOX* gene expression might also allow patients to be stratified according to their likely response to such a treatment. We hope that further work will help us to refine this approach to establish the selective activation of necroptosis as a therapeutic target in AML.

## MATERIALS AND METHODS

### Analysis of AML microarray data

The bioinformaritcs analysis was performed in R. The GSE23312 microarray data set [30] was downloaded from the GEO database [11] and pre-processed in R/Bioconductor [31]. The patients were stratified equally into 2 groups based on the expression levels of each *HOX* gene. The association between the expression level and patient survival was assessed using Kaplan-Meier plots in R using the survival package.

### Primary AML cell isolation

Primary AML cells were obtained after patient's informed consent and approval of the study by the ethics committee (SGUL). Cells were isolated from bone marrow or peripheral blood using density gradient centrifugation and cultured in IMDM medium (Life Technologies, Carlsbad, CA, USA) with 10% foetal bovine serum (FBS; Biochrom GmbH, Berlin, Germany), 10% horse serum (HS; Gibco, Invitrogen, Carlsbad, USA), and 1 μM hydrocortisone (Sigma-Aldrich, St. Louis, MO).

### Proliferation assays

For adaptation to oxygen content, primary AML cells were cultured for three days under hypoxic conditions and then seeded for proliferation assays. The cells were subsequently incubated with different concentrations of HXR9 or CXR9 ranging from 100 nM to 5000 nM. All assays were done in triplicate. Cell numbers were determined on day 3 after HXR9/CXR9 treatment using the cell viability analyzer Vi-Cell XR (Beckman Coulter, Brea, CA, USA). Each assay was performed at least twice.

### P21 ELISA

P21 protein was measured using the p21 simplestep ELISA kit (Abcam, Cambridge, UK) according to the manufacturer's instructions.

### AML derived cell lines

In this study we used three primary AML cell lines derived from patients with *de novo* mutations: KG-1, a cell line derived from an erythroleukaemia [32], HEL 92.1.7, an erythroleukaemia cell line [33], and HL-60, a cell line obtained from acute promyelocytic leukaemia (APML) [34]. The two other lines, K562 (26) and KU812F (27) were derived from secondary AML (chronic myeloid leukaemia cells in blast crisis).

### HXR9 and CXR9 synthesis

The synthesis of these peptides has been described previously [35].

### Tissue culture

K562, KU812F and HEL92.1.7 cells were grown in RPMI-1640 medium complemented with 10% FBS, 5% L-glutamine and 5% antibiotics (1000 units of penicillin/ml and 10 mg/ml streptomycin, P/S). KG-1 and HL-60 cells were cultured in IMDM supplemented with 20% FBS and 5% antibiotics. All cell lines were cultured in T-75 sterile cell culture flasks at 37°C in a humidified environment containing 5% CO_2_ and 95% air.

### Gene expression analysis by RT-PCR

Gene expression analysis using SYBR green^TM^ was carried out in order to measure the changes in expression of genes of interest. The primer sequences for the pro- and anti-apoptotic genes are listed in Table 1. Each gene of interest was tested in triplicate. The housekeeping gene *β-actin* was used as an endogenous control. The thermal profiles employed were: heating once for 10 minutes at 95°C, followed by 30 seconds at 95°C, 1 minute at 60°C, 30 seconds at 72°C for 40 cycles. The mean of the cycle threshold values of the triplicate repeats for each gene was calculated and used for measuring gene expression.

**Table 1:** Pro- and anti-apoptotic and *β-actin* gene primers used for PCR amplification

**Gene**	**Forward Sequence 5’-3’**	**Reverse Sequence 5’-3’**	**Amplicon Length (bp)**
***Bad***	CGGAGGATGAGTGACGAGTT	GATGTGGAGCGAAGGTCACT	180
***Bak1***	TTTTCCGCAGCTACGTTTTT	GGTGGCAATCTTGGTGAAGT	248
***Bax***	TTTGCTTCAGGGTTTCATCC	CAGTTGAAGTTGCCGTCAGE	246
***Bcl2***	GAGGATTGTGGCCTTCTTTG	ACAGTTCCACAAAGGCATCC	170
***Bid***	CTGCAGGCCTACCCTAGAGA	ACTGTCCGTTCAGTCCATCC	195
***XIAP***	GGGGTTCAGTTTCAAGGACA	CGCCTTAGCTGCTCTTCAGT	182
***Apaf1***	TTCTGATGCTTCGCAAACAC	CTGGCAAATCTGCCTTCTTC	237
***Caspase-3***	TTTTTCAGAGGGGATCGTTG	CGGCCTCCACTGGTATTTTA	151
***Caspase-6***	ATCCTCACCGGGAAACTGTG	AATTGCACTTGGGTCTTTGC	161
***Caspase-7***	AGTGACAGGTATGGGCGTTC	CGGCATTTGTATGGTCCTCT	164
***Caspase-9***	CTAGTTTGCCCACACCCAGT	GCATTAGCGACCCTAAGCAG	172
***PARP1***	GCTCCTGAACAATGCAGACA	CATTGTGTGTGGTTGCATGA	233
***c-FOS***	CCAACCTGCTGAAGGAGAAG	GCTGCTGATGCTCTTGACAG	232
***p21***	GACACCACTGGAGGGTGACT	CAGGTCCACATGGTCTTCCT	171
***p53***	GTGGAAGGAAATTTGCGTG	CCAGTGTGATGATGGTGAGG	183
***β-actin***	ATGTACCCTGGCATTGCCGAC	GACTCGTCATACTCCTGCTTG	227

### Lactate dehydrogenase (LDH) assay

Cells were suspended in 5% FBS of the appropriate culture medium at a concentration of 5×10^5^ cell/ml and seeded in 96-well flat-bottom plates at a concentration of 5×10^4^ cell/well. The cells were then treated with 100 μl of a serial dilution of HXR9 or its negative control CXR9 in 5% FBS for 2 hours. Each plate included untreated cells (negative control), 2% triton-treated cells (positive control), and 5% FBS medium only (background control). LDH enzymatic activity was determined according to the manufacturer's instructions.

### Annexin V- PE assay

The annexin V-PE assay was used to quantify the percentage of the apoptotic cells and evaluate the mechanism of cell death. Cells were suspended in 5% FBS of the appropriate culture medium at a concentration of 1×10^6^ cell/ml and seeded in 96-well flat-bottom plates at a concentration of 1×10^5^ cell/well. The percentage of the different cell populations, viable, early apoptotic and dead cells, were plotted by GraphPad Prism software (California, USA) on column graphs to allow the comparison of treated and untreated negative control cells. Each experiment was repeated at least 3 times independently.

### DAPI staining

Cells were suspended in 5% FBS culture medium at a concentration of 1×10^6^ cell/ml and seeded in 24-well plates at a concentration of 5×10^5^ cell/well. Then cells were treated either with HXR9 at the IC_50_ or double the IC_50_, in duplicate. Each plate included untreated negative control cells.

### Cyclosporin A (CsA) protection assay

CsA attenuates mitochondrial permeability transition pore (mPTP) formation by binding to cyclophilin D (cypD), which is required for mPTP formation in necrotic mitochondrial cell death [36–38]. In this assay cells were pre- and co-treated with or without 5 μM CsA and hydrogen peroxide (H_2_O_2_) treatment was used as a positive control. Cells were treated in triplicate with HXR9 at the IC_50_, double or triple the IC_50_ or with 20 mM H_2_O_2_. Cell pellets were prepared and stained with annexinV-PE as described for the annexinV-PE assay. The proportion of positively stained cells was determined using flow cytometry.

### Necrostatin-1 (Nec-1) protection assay

Nec-1 is a selective inhibitor of receptor interacting protein 1 (RIP1) [39]. RIP1 inhibition prevents a form of programmed necrosis called necroptosis [40]. Cells were pre- and co-treated with 50 μM Nec-1 using the same method described above for the CsA protection assay.

### Fructose protection assay

Fructose is a glycolytic substrate that at high concentrations significantly sequesters phosphate and accordingly intracellular ATP is depleted. This selective depletion of ATP protects cells from apoptosis, but leaves sufficient ATP to rescue cells from spontaneous necrosis [41]. Cells were treated with HXR9 or with 2% triton as a positive control and cell viability was measured using the LDH assay as described above.

### Ethilenediaminetetra-acetic acid (EDTA) and HXR9 cytotoxicity

The role of Ca^2+^ in HXR9-mediated cell death was assessed by two different assays, LDH and annexin V, as described for the fructose protection and CsA protection assays, respectively.

### Inhibition of mitogen activated protein kinase (MAPK) pathways

The effect of HXR9 on MAPK pathways was studied using specific inhibitors of extracellular signal-regulated kinase (ERK), Jun N-terminal kinase (JNK), and p38 [42], which were U0126 monoethanolate, SP600125, and SB203580, respectively. Experiments were performed as described for the fructose protection assay.

### NADPH oxidase (NOX) inhibition

NOX is a generator of intracellular reactive oxygen species (ROS) [43]. The effects of NOX on the efficiency of HXR9 were assessed using the NOX inhibitor diphenyleneiodonium chloride (DPI) [44]. Cells were pre- and co-treated with or without 60 μM DPI. The assessment of DPI effect on the cytotoxicity of HXR9 was performed as described for the fructose protection assay.

### μ-calpain inhibition

Calpains are Ca^2+^-dependent cysteine proteases that commonly activated in Ca^2+^ dependent cell death mechanisms [45]. Among them, μ-calpain is one of the most extensively studied in apoptosis/necrosis processes. Calpain inhibitor I (**N-Acetyl-Leu-Leu-Norleu-al) was used to inhibit** the Ca^2+^/μ-calpain pathway. Cells were pre- and co-treated with or without 60 μM calpain inhibitor I. The assessment of the role of μ-calpain in the cytotoxicity of HXR9 was performed as described for the fructose protection assay.

### PKC inhibition

Ro31-8220 (methanesulfonate salt) was used to inhibit and assess the role of PKC in HXR9 cytotoxicity. Cells were pre- and co-treated with or without 30 μM Ro31-8220, and its effects on the cytotoxicity of HXR9 was assessed as for the fructose protection assay.

### Heme oxygenase-1 (HO-1) inhibition

HO-1 is an anti-apoptotic protein [46]. Protoporphyrin IX (PPIX) was used to inhibit HO-1 protein and assess its role in the cytotoxicity of HXR9. Cells were pre- and co-treated with or without 50 μM of PPIX, and the effect of HO-1 inhibition on HXR9 cytotoxicity was determined as described for the fructose protection assay.

### p53 inhibition

Pifithrin-α (PFT-α) is an inhibitor of p53-dependent apoptosis and was used to block and assess the role of p53 in the cytotoxicity of HXR9. Cells were pre- and co-treated with or without 40 μM of PFT-α, and the role of p53 in HXR9 cytotoxicity was assessed as described for the fructose protection assay.

### *In vivo* model

Female C57BL/6, nude and severe combined immune-deficient mice, approximately 6 to 8 weeks old, were purchased from Jackson laboratory, Kent, UK. These mice were maintained in pathogen-free cages and fed with irradiated food and acidified water. All experiments were performed under the compliance of Home Office and institutional instructions. The mice were anesthetised using isoflurane and K562 cells (5×10^6^ cell/mouse) were then injected subcutaneously into the flank. Flank tumour sizes were measured three times a week using calliper measurement. Tumour sizes were calculated using the formula: (length x width x width)/2. When the flank tumour became palpable, the mice were divided into two groups with one treated intratumourly with 50 μl of the indicated dose of HXR9, CXR9, Ro31, HXR9 combined with HXR9, or with PBS alone (control group). Mice were sacrificed if the tumour volume was ≥ 1500 mm^3^.

### Statistical analysis

Calcusyn software was used to determine the IC_50_ of the different drugs for the different cell lines and for assessing whether the interaction between HXR9 with DNR or MTX was synergistic, additive or antagonistic, as described below. Student's *t-test* was applied for data analysis of all assays using GraPhpad Prism software. Results are expressed as the mean of 3 separate experiments with error bars to show the SEM. Statistical significance was determined by comparing the different conditions in each assay with each other and considered significant when *p* < 0.05 (*), *p* < 0.01 (**), *p* < 0.001 (***), or *p* < 0.0001 (****). The IC_50_ of HXR9, DNR, and MTX for different cell lines were calculated by the median-effect equation of Chou [47] using Calcusyn software (Biosoft, Cambridge, UK). The combination effects of HXR9 with either DNR or MTX were calculated using the combination index (CI) equation [48] with Calcusyn Software. CI > 1, CI = 1, and CI < 1 indicate antagonism, an additive effect and synergism, respectively.

## Abbreviations

AML: acute myeloid leukemia
APML: acute promyelocytic leukaemia
CLL: chronic lymphocytic leukaemia
CsA: cyclosporin A
cypD: cyclophilin D
cytC: cytochrome C
DPI: diphenyleneiodonium chloride
EDTA: ethilenediaminetetra-acetic acid
FBS: foetal bovine serum
GEO: gene expression omnibus
HO-1: heme oxygenase-1
HS: horse serum
LDH: lactate dehydrogenase
MAPK: mitogen activated protein kinase
mPTP: mitochondrial permeability transition pore
Nec-1: necrostatin-1
NOX: NADPH oxidase
PFT-α: pifithrin-α
PKC: protein kinase C
PPIX: protoporphyrin IX
RIP1: receptor interacting protein 1
TNFR-1: tumour necrosis factor receptor-1
TRADD: TNFR-1-associated death domain protein
